# Editorial: RNA editing and modification in development and diseases

**DOI:** 10.3389/fgene.2022.1025445

**Published:** 2022-10-04

**Authors:** Yanqiang Li, Jia Meng, Dongyu Zhao

**Affiliations:** ^1^ Basic and Translational Research Division, Department of Cardiology, Boston Children’s Hospital, Boston, MA, United States; ^2^ Department of Pediatrics, Harvard Medical School, Boston, MA, United States; ^3^ Department of Biological Sciences, Xi’an Jiaotong-Liverpool University, Suzhou, Jiangsu, China; ^4^ Institute of Systems, Molecular and Integrative Biology, University of Liverpool, Liverpool, United Kingdom; ^5^ AI University Research Centre, Xi’an Jiaotong-Liverpool University, Suzhou, Jiangsu, China; ^6^ Computational Biology and Bioinformatics at the School of Basic Medical Sciences, Peking University, Beijing, China

**Keywords:** RNA modification, m6A, m5C, RNA binding protein, RNA editing

RNA editing is a critical co-/post-transcriptional process perturbing the RNA in eukaryotic cells. RNA editing could occur in coding and non-coding regions, affecting the recoding of proteins, RNA splicing and stability. Adenosine to inosine (A-to-I) editing catalyzed by adenosine deaminases acting on RNA (ADAR) is the most dominant (∼90%) RNA editing event in mammals (Athanasiadis et al., 2004). Despite extensive studies of RNA editing in human tissues, cancer development (Han et al., 2015) and the neural system (Hoopengardner et al., 2003), the study of RNA editing in other species apart from human and mouse were still limited. On the other side, the regulation of RNA editing in diseases such as cardiovascular, immune, and metabolism disorders needs to be investigated (Uchida and Jones, 2018). Besides RNA editing, general RNA modifications, including, m6A (Dominissini et al., 2012), pseudouridine (Carlile et al., 2014), m5C (Edelheit et al., 2013), m1A (Safra et al., 2017) and 2′-O-methylation (Dai et al., 2017; Elliott et al., 2019; Yi et al., 2021), have emerged as a critical layer for gene expression regulation, known as the epitranscriptome, attracting the best scientists globally.

To understand the role of m6A modification in lung cancer, Ma and Zhang conducted a consensus clustering analysis of 502 lung adenocarcinoma (LUAD) samples from TCGA based on the expression profiles of 20 m6A regulators. They found two m6A modification patterns with distinct overall survival (OS), activation of signaling pathways and tumor immunity. Furthermore, they identified 213 prognostic m6A-related genes, which were imported into LASSO-cox regression analysis. Next, they developed the m6A risk score and found that patients with low m6A risk score exhibited a prominent survival advantage in an independent dataset. Finally, they established a highly accurate nomogram containing independent prognostic indicators. N6-methyladenosine (m6A) modification plays an important role in regulating the immunity microenvironment of breast cancer (BRCA). Zhang et al. established an m6A-related immune score (m6A-IS) to predict the immune infiltration and prognosis of BRCA accurately. The Low m6A-IS group is associated with enhanced antigen presentation and improved immune checkpoint expression, thus indicating sensitivity to immunotherapy. Furthermore, the m6A-IS can independently predict BRCA patients’ response to immunotherapy.

Yang et al. attempted to map the m6A epitranscriptome of neuromyelitis optica spectrum disorder (NMOSD) patients compared to healthy controls. Towards this blood samples of NMOSD patients and healthy controls, the RNA were isolated and subjected to m6A-seq followed by bioinformatic analysis. Authors *via* bioinformatic analysis highlighted an extensive list of hyper and hypo m6A methylated transcripts and have attempted to connect it to NMOSD pathophysiology.

Ma et al. conducted an m6A-related lncRNA prognostic model using TCGA data. Twelve lncRNAs were included and validated in their new model. Also, the model was internally validated and showed good predictive values for ccRCC’s survival.

Huang et al. studied breast cancer (BC)-specific m5C-related lncRNAs (m5C-lncRNAs) as potential biomarkers in breast cancer. They made a prognostic risk model, and analyzed the characteristics of tumor-infiltrating immune cells based on the subtypes of the risk model. The study used *in silico* data from TCGA database and for a validation experiment (sixteen pairs of fresh BC and paracarcinoma tissues) from their own patients. They found that these lncRNAs may serve as prognostic biomarkers in breasr cancer.

He et al. downloaded harmonized RNA-seq count data and clinical data for AML from several large study cohorts and analyzed the differential expression of a set of RNA binding proteins (RBPs) using the R package edgeR software together with statistical methods including univariate Cox regression analysis, LASSO-Cox regression analysis and multivariate Cox regression analysis. The authors established a prognostic model of 12-RBPs gene for AML and C-index and calibration diagrams were used to judge the accuracy of the model, and DCA was used to judge the net benefit. They have found that the net benefit and prediction accuracy of the prognostic model and the mixed model based on it was significantly higher than that of cytogenetics which was verified in one of the study cohorts where the data collected from and both of the selected gene set and the LASSO results have high credibility. By that, the authors concluded that their prognosis model of 12-RBPS gene is an optimized biomarker that can effectively stratify the risk of AML patients and the nomogram based on this prognostic model is a reliable method to predict the median survival time of patients.

Satir-Basaran et al. explored the role of ncRNA as vector for epigenetic inheritance *via* paternal germline. In their experimental set-up, the authors use four lines of mice (two susceptible to diet-induced obesity and diabetes and two resistant) and then perform phenotypic analyses in the males directly exposed to the diet, as well as in the following two generations of mice obtained *via* paternal transmission. To support a role for ncRNA, the authors rely on microinjection of RNA into fertilized oocytes. They used synthetic miR-19b-5p (found previously to induce obesity and diabetes), total sperm RNA, or RNA that was fractionated based on size (small, <200 nt and long, >200 nt) or subcellular location (DNA bound and free), which were obtained from males exposed to normal diet or diets with excess of fat. Overall, they conclude that ncRNA plays a role in the inter/trans-generational inheritance of the phenotypes and propose that the DNA-bound RNA may be a critical component.

Luo et al. investigated the expression of pyroptosis related genes in colon adenocarcinoma (COAD) and build a risk score model by utilizing the differential expressed genes. The model was found effective in predicting the overall survival and prognosis of COAD patients.

This review by Hao et al.gave an introduction about RNA editing, including RNA editing forms and organelles RNA editing occurred, then summarized the factors and mechanism involved in RNA editing in plant organelles, further reviewed RNA editing events identified in plant organelles through deep sequencing data, and finally discussed the functions of RNA editing in plant organelles. Pentatricopeptide repeat (PPR) proteins, one type of RNA-binding protein, are particularly prevalent in land plants. It has been revealed that PPR proteins are involved in the RNA processing of organellar genes and play a vital role in plant development and defense. In this manuscript, Qin et al. summarizes the recent progress in functional studies on PPR proteins, including plant fertility, chloroplast biogenesis, embryogenesis, stress responses, and plant development.

Li et al. performed univariate analysis to identify the prognosis-related RNA binding proteins (RBPs). They identified 11 RBPs as prognosis markers of HBV-related hepatocellular carcinoma (HCC). In addition, the authors claimed that their model has better predictive efficacy than the models using other clinical parameters.

Although lot of studies of RNA modification and editing in the development and diseases, there are several new aspects need further exploration. Because there are more than 140 kind of RNA modifications in RNA, new methods to detect novel RNA modification systemically are needed to develop. E.g Deep learning based method (Song et al., 2021) and new non-antibody technology will be good to find the RNA modification at base-resolution (Hu et al., 2022); On the otherside, with development of single cell technology, study RNA modification/editing at the single cell levels or even spatial levels will be helpful to understand the cellular development with a high resolution (Sapiro et al., 2019); In addition, intergration of the GWAS data of specific disease to see the mutation the RNA binding proteins related to the RNA modification will be helpful to reveal the role of RNA modifications in disease (Nachmani et al., 2019).

To summarize, our unique topic covers RNA modifications such as m6A and m5C in disease, the role of RNA binding proteins and non-coding RNAs, and the editing progress of RNA editing in plants. These research studies are complemented by review articles that summarize our current understanding of RNA modifications in human disease and development. We hope our Research Topic will benefit the community of RNA modifications.
